# Supportive Housing Program and Influenza Vaccination Rates Among Veterans Experiencing Homelessness

**DOI:** 10.1001/jamanetworkopen.2026.0001

**Published:** 2026-02-24

**Authors:** Laura A. Graham, Hannah C. Decker, Jack Tsai

**Affiliations:** 1 Health Economics Resource Center, Veterans Affairs Palo Alto Health Care System, Menlo Park, California; 2 S-SPIRE, Stanford University, Stanford, California; 3 Department of Surgery, University of California, San Francisco, San Francisco; 4 National Center on Homelessness Among Veterans, VA Homeless Programs Office, Washington, DC; 5 School of Public Health, University of Texas Health Science Center at Houston, Houston

## Abstract

This cohort study estimates the association of participation in a supportive housing program with influenza vaccination rates among veterans experiencing homelessness.

## Introduction

People experiencing homelessness face major barriers to accessing preventive health services, such as annual influenza vaccinations.^1,2^ As a result, this group has lower rates of preventive service uptake despite being at higher risk for infectious diseases.^3^ Programs like Department of Housing and Urban Development–Veterans Affairs Supportive Housing (HUD-VASH) help overcome barriers by providing stable housing and connecting participants to Veterans Health Administration (VHA) services. The program offers Housing Choice Vouchers, VHA case management, and additional supportive services for eligible veterans.

We hypothesized that HUD-VASH’s housing and supportive services would be associated with increased preventive service use. Our goal was to estimate the association of HUD-VASH participation with influenza vaccination rates at the start of influenza season, compared with veterans in VHA care experiencing homelessness and not enrolled in HUD-VASH.

## Methods

This is a retrospective cohort study of veterans experiencing homelessness receiving care through VHA. The study was reviewed and approved by the Stanford institutional review board with a waiver of informed consent. The manuscript was drafted in accordance with the current STROBE reporting guidelines. Veterans were included if they were identified as experiencing homelessness for at least 2 consecutive months between June 1, 2020, and November 30, 2024.

Housing status was defined using previously described methods, including data from VHA HOMES and the Corporate Data Warehouse (CDW).^4,5^ Influenza vaccinations were identified from the CDW Immunization, Inpatient, Outpatient, and Bar Code Medication Administration tables. Patient demographics, comorbidity burden, and health care utilization at the first instance of homelessness were obtained from the CDW.

We used χ^2^ tests and *t* tests to examine bivariate differences. A longitudinal differences-in-differences analysis, where each participant serves as their own control, was used to evaluate the association between HUD-VASH and vaccination. The treatment period was defined as HUD-VASH program enrollment from June 1 to August 31 of each calendar year during the study period. The outcome was influenza vaccination during the subsequent 3 months, September 1 to November 30 (follow-up period), which aligns with the peak influenza vaccination season. The comparison group is veterans experiencing homelessness who did not participate in the HUD-VASH program. Logistic regression, excluding incomplete cases, was used to estimate the association of HUD-VASH with influenza vaccination. Statistical significance was set at 2-sided *P* < .05. Data were analyzed using R statistical software version 4.5.2 (R Project for Statistical Computing). Additional details on the assumptions of our differences-in-differences design are included in the eAppendix in Supplement 1.

## Results

We identified 497 892 veterans experiencing homelessness, of whom 61 018 (12.3%) entered the HUD-VASH program between June 1 and August 31 each year of the study. The study population included 434 207 male veterans (87.5%), with a mean (SD) age of 52 (16) years (Table 1). A total of 110 493 patients (22.2%) were vaccinated during their 3-month follow-up (19 587 HUD-VASH enrollees [32.1%] vs 90 915 other veterans experiencing homelessness [20.8%]; standardized mean difference, 0.258; 95% CI, 0.249 to 0.266; *P* < .001). Influenza vaccinations were more common among older veterans (58 vs 52 years) and veterans with more comorbidities at the start of the treatment period. Assuming parallel trends and adjusting for participant characteristics, veterans recently enrolled in HUD-VASH had 48% higher odds of receiving an influenza vaccine than other veterans experiencing homelessness (odds ratio, 1.48; 95% CI, 1.19-1.85; *P* < .001) (Table 2).

**Table 1.** Characteristics of the Study Participants by HUD-VASH Status

Characteristic	Participants, No. (%)			SMD (95% CI)^a^
	Overall (N = 497 892)	HUD-VASH (n = 61 018 [12.3%])	No HUD-VASH (n = 436 874 [87.7%])	
Any influenza vaccination				
Yes	110 493 (22.2)	19 578 (32.1)	90 915 (20.8)	0.258 (0.249 to 0.266)
No	387 399 (77.8)	41 440 (67.9)	345 959 (79.2)	
Sex				
Female	62 121 (12.5)	8081 (13.3)	54 040 (12.4)	0.025 (0.017 to 0.034)
Male	434 207 (87.5)	52 901 (86.7)	381 306 (87.6)	
Missing^b^	1564	36	1528	
Self-reported race				
Black or African American	160 754 (37.0)	21 483 (40.3)	139 271 (36.5)	0.094 (0.085 to 0.103)
White	251 056 (57.7)	28 716 (53.8)	222 340 (58.3)	
Other^c^	23 190 (4.7)	3149 (5.6)	20 041 (5.2)	
Missing^b^	62 892	7670	55 222	
Patient age, mean (SD), y	52 (16)	51 (14)	52 (17)	−0.687 (−0.810 to −0.563)
Missing	14	0	14	
Marital status				
Divorced	152 340 (32.5)	20 477 (35.8)	131 863 (32.0)	0.248 (0.239 to 0.257)
Married	121 072 (25.8)	9750 (17.0)	111 322 (27.0)	
Never married	145 624 (31.1)	20 618 (36.0)	125 006 (30.4)	
Separated	35 079 (7.5)	4724 (8.3)	30 355 (7.4)	
Widowed	14 695 (3.1)	1631 (2.9)	13 064 (3.2)	
Missing^b^	29 082	3818	25 264	
Charlson Comorbidity Index score, mean (SD)	1.15 (2.19)	0.90 (1.81)	1.15 (2.20)	−0.285 (−0.303 to −0.267)
Unknown	43 506	67	43 439	
Prior primary care use within 2 y	329 812 (66.2)	34 517 (56.6)	295 295 (67.6)	−0.110 (−0.114 to −0.106)

Abbreviations: HUD-VASH, Department of Housing and Urban Development–Veterans Affairs Supportive Housing; SMD, standardized mean difference.

^a^ Welch 2-sample *t* test or 2-sample test for equality of proportions was used with continuity correction. All *P* < .001.

^b^ Missing data were not included in calculations of percentages.

^c^ Other race includes American Indian, Alaska Native, Asian, Native Hawaiian, Other Pacific Islander, and patients who self-identified as multiple races during the study period.

**Table 2.** Differences-in-Differences Model of the Odds of Receiving an Influenza Vaccination Among Veterans Experiencing Homelessness

Variable	OR (95% CI)^a^	*P* value
Treatment		
Not enrolled in HUD-VASH	1 [Reference]	<.001
Enrolled in HUD-VASH	1.49 (1.20-1.85)	
Time		
June-August	1 [Reference]	<.001
September-November	23.82 (22.89-24.80)	
Treatment by time interaction	1.48 (1.19-1.85)	<.001

Abbreviations: HUD-VASH, Department of Housing and Urban Development–Veterans Affairs Supportive Housing; OR, odds ratio.

^a^ Adjusted for age, sex, race (including a category for missing race), marital status, calendar year, comorbidity burden as measured by the Charlson Comorbidity Index, and use of Veterans Affairs primary care in the year prior.

## Discussion

Multicomponent interventions like HUD-VASH can improve preventive service uptake among homeless populations by helping individuals prioritize health and reduce barriers to care.^6^ Limitations of this cohort study include unmeasured confounding between groups, potential misclassification bias of housing status, self-selection into HUD-VASH, and differences in housing and service contexts by site. Differences in health care–seeking behavior, seasonal vaccination promotion programs, access to care, or varying levels of social support and engagement may also influence both program participation and likelihood of receiving an influenza vaccination. These findings underscore the promise of integrated housing and health care strategies for improving preventive health in people experiencing homelessness.

## Notes

Supplement 1. eAppendix. Assumptions of the Differences-in-Differences (DiD) Design

Supplement 2. Data Sharing Statement
