# Supplementary material for: Supportive Housing Program and Influenza Vaccination Rates Among Veterans Experiencing Homelessness
Source: JAMA Netw Open. 2026 Feb 24;9(2):e260001. doi: 10.1001/jamanetworkopen.2026.0001 (PMC12933278; doi:10.1001/jamanetworkopen.2026.0001)
Supplement: Supplement 1. — eAppendix. Assumptions of the Differences-in-Differences (DiD) Design [file jamanetwopen-e260001-s001.pdf]

Supplemental Online Content

Graham LA, Decker HC, Tsai J. Supportive housing program and influenza vaccination rates among veterans experiencing homelessness. *JAMA Netw Open*. 2026;9(2):e260001. doi:10.1001/jamanetworkopen.2026.0001

eAppendix. Assumptions of the Differences-in-Differences (DiD) Design

This supplemental material has been provided by the authors to give readers additional information about their work.

© 2026 Graham LA et al. *JAMA Network Open*.

eAppendix. Assumptions of the Differences-in-Differences (DiD) Design

Parallel Trends Assumption: In the absence of the intervention (HUD-VASH enrollment), the average change in flu vaccination rates over time would have been the same for both the treatment group (HUD-VASH enrolled) and the control group (not HUD-VASH enrolled). Any difference in trends between the groups after the intervention is attributed to the effect of the intervention, not to pre-existing differences or other factors.

To assess this assumption, we compared trends of flu vaccination in the pre-intervention period (June-August). Given the large sample size, visual inspection was used, and we concluded that the parallel trends assumption was not violated. We also included an adjustment for age, sex, race, marital status, comorbidity burden as measured by the Charlson Comorbidity Index, and use of VA primary care in the year prior in the final DiD model.

No Simultaneous Interventions (No Confounding Events): We have no reason to believe that any other events or interventions occurred during the study period that differentially affected the vaccination rates of the treatment and control groups. This ensures that observed changes are due to HUD-VASH enrollment and not to other policies or shocks that might coincide with the intervention and differentially affect the treatment and control groups.

Stable Unit Treatment Value Assumption (SUTVA): We have no reason to believe that the HUD-VASH enrollment of one individual affects the outcomes of another (i.e., there are no spillover effects). Each subject's likelihood of receiving a flu vaccination between September and November depends only on their own treatment status, not on the treatment status of others in the study.

Composition Stability: To alleviate concerns about loss to follow-up, we have limited our pre- and post-periods to 3 months, so there should be no differential attrition or group membership change that could bias the results (e.g., people moving between groups or dropping out at different rates).

© 2026 Graham LA et al. *JAMA Network Open*.
