# Supplementary material for: Supportive Housing Program and Influenza Vaccination Rates Among Veterans Experiencing Homelessness
Source: JAMA Netw Open. 2026 Feb 24;9(2):e260001. doi: 10.1001/jamanetworkopen.2026.0001 (PMC12933278; doi:10.1001/jamanetworkopen.2026.0001)
Supplement: Supplement 2. — Data Sharing Statement [file jamanetwopen-e260001-s002.pdf]

Data Sharing Statement

Graham. Supportive Housing Program and Influenza Vaccination Rates Among Veterans Experiencing Homelessness. *JAMA Netw Open*. Published February 24, 2026. doi:10.1001/jamanetworkopen.2026.0001

Data The dataset contains sensitive information that restricts sharing in accordance with institutional policies and ethical guidelines pertaining to confidentiality and participant privacy. Access to the data is limited to the research team, and any requests for data access will be considered on a case-by-case basis in compliance with these guidelines. For inquiries regarding the data or potential collaboration, please contact the corresponding author.
Data available: No
